# Cocaine Effects on Reproductive Behavior and Fertility: An Overview

**DOI:** 10.3390/vetsci10080484

**Published:** 2023-07-25

**Authors:** Luigi Rosati, Teresa Chianese, Aldo Mileo, Maria De Falco, Anna Capaldo

**Affiliations:** 1 Department of Biology, University Federico II, Via Cinthia 21, 80126 Naples, Italy; teresa.chianese2@unina.it (T.C.); aldo.mileo@unina.it (A.M.); madefalco@unina.it (M.D.F.); anna.capaldo@unina.it (A.C.); 2 Centro Interdipartimentale di Ricerca “Ambiente” (CIRAM), University Federico II, 80134 Naples, Italy; 3 Istituto Nazionale Biostrutture e Biosistemi (INBB), 00136 Rome, Italy

**Keywords:** drug, cocaine, testis, ovary, reproduction, sex hormones, hormonal control

## Abstract

**Simple Summary:**

Cocaine, a widely used drug, can influence sexual behavior. It has been shown, especially in mammals, that cocaine can cause various disorders of sexual activity and gonadal dysfunction in both sexes. Cocaine has been found to alter the cell cycle, alter the meiotic spindle, induce apoptosis, and alter sperm motility. This review provides an overview of the main effects of cocaine on spermatogenesis and oogenesis, laying the groundwork for future investigations into the molecular mechanisms underlying the interaction of cocaine and its metabolites with germ cells.

**Abstract:**

Cocaine is one of the most widely used drugs that, due to its molecular properties, causes various behavioral alterations, including sexual behavior. In vivo and in vitro studies conducted mainly in mammals have shown various disorders of sexual activity and morpho-functional dysfunctions of the gonads in both sexes. Although the modalities are still unclear, cocaine has been shown to alter the cell cycle, induce apoptosis, and alter sperm motility. In females, this drug alters the formation of the meiotic spindle as well as may obstruct the ovulation mechanism of mature oocytes. The data provided in this review, in addition to reviewing the current literature on the main effects of cocaine on spermatogenesis and oogenesis mainly in mammals, will hopefully provide a basic overview that may help and support further future studies on the molecular interaction of cocaine and its metabolites with germ cells.

## 1. Introduction

### 1.1. Cocaine Production and Use

Drug dependence is a global problem, responsible for enormous health damage; it is estimated that 230, 93, and 31 million years of “healthy” life were lost in 2019 due to tobacco, alcohol, and drug addiction, respectively [1]. Among the most used drugs is cocaine, and its production reached a high record in 2020 of 1982 tons, with an increase of 11% compared to the previous year. It was estimated in 2020 that approximately 2.5 million people aged 15–64 used cocaine at least once in the past year, representing 0.4% of the global population, with an increase in the number of users compared to 2010 [2]. This is alarming if we consider that stimulant drugs of the cocaine type are considered responsible for the highest number of drug-related deaths in 11% of 48 member states reporting qualitative assessments to the UNODC [3].

Cocaine is a psychostimulant substance of plant origin, mainly obtained from the species *Eritroxylum coca*, native to the tropical forests of the eastern Peruvian Andes (Bolivia, Peru, Ecuador). The youngest leaves of this plant contain about 1% of cocaine and are used to produce the drug; the leaves deteriorate very easily in humid environments; therefore, the alkaloid is usually extracted in the places of collection before the export. Although there are several ways to prepare cocaine, usually, this process requires three successive stages, the first of which involves the extraction of crude coca paste from the coca leaf. The second stage involves the purification of coca paste to coke base, which is then converted to cocaine hydrochloride [4], the formulation of cocaine mainly used and historically best known. From cocaine hydrochloride, it is possible to prepare two other known forms of cocaine that make the substance smokeable: basic cocaine or crack and freebase cocaine. These are both free bases because they are not salified with hydrochloric acid but are of different “purity”, and cocaine base or crack contains many impurities, diluents, and adulterants. For example, if an excess of ammonia (NH_3_) is used for the preparation of a crack, and it has not been washed and dried off before a crack is smoked, it may contain residues of this substance, which is acutely toxic and will damage lips, mouths, windpipes, and lungs. Freebase cocaine is instead a purer form of cocaine than crack, but the grade of purity depends largely on the purity of cocaine hydrochloride used as a starting material and on the organic solvent used. Finally, it should be borne in mind that all powders sold on the illicit market as cocaine hydrochloride are never 100%, but always contain different substances in varying quantities, mainly impurities and cutting agents, such as diluents and adulterants, which may increase the toxicity of cocaine [5].

Cocaine has mainly three types of effects: (1) it acts as a local anesthetic because of its ability to block neural conduction by binding to sodium channels; (2) it is a sympathomimetic drug, in fact, increases heart rate and blood pressure, and causes vasoconstriction and hyperthermia; (3) it is a stimulant of the central nervous system (CNS) and thus increases alertness, induces euphoria, sharpens perceptual sensations, gives a sense of increased physical strength and mental capacity, and decreases the feeling of sleep and hunger. The effects of cocaine occur quickly and depend on the mode of intake; psychological effects are extremely variable in nature as they depend on emotional states and expectations and are related to the intake of the substance and the credited value to cocaine [6]. The effects usually sought are increased alertness and mental abilities, euphoria, increased perceptive sensations, confidence, self-confidence, increased physical strength and resistance to fatigue, as well as increased intensity of sexual emotions and sensations [6]. The objective of this review is to analyze the current literature related to the effects of cocaine on the sexual component, paying particular attention to possible dysfunction in males and females of both sexual behavior and gonadal function.

### 1.2. Cocaine Pharmacokinetics and Pharmacodynamics

The effects of cocaine heavily depend on the administration method. Chewing coca leaves, often in the presence of an alkaloid that increases absorption through the oral mucosa, is a mode of intake that does not lead to addiction or overdose, as cocaine is slowly absorbed through the gastrointestinal tract and accumulates slowly and in small doses in the brain [7]. Similarly, cocaine, being an alkaloid, is not well absorbed when administered orally due to the secretions of the gastrointestinal tract. Cocaine hydrochloride is commonly self-administered by sniffing it (snorted) so that it is absorbed through the nasal mucosa; this route ensures a faster onset of action and higher brain drug concentration than oral administration, in turn, related to the possibility of developing dependence. However, sniffing leads to tissue degeneration caused by ischemia related to the vasoconstrictive properties of cocaine [7]. These same properties are responsible for the fact that when the drug is snorted, the absorption is slower and the effects more lasting than when the drug is smoked. Coca paste can be smoked; it has high organic and neurological toxicity due to the presence of many of the substances used in its processing, mainly solvent residues, which can be inhaled as combustion products [7]. Finally, coca paste also has a remarkable addictive ability. However, the main form that is smoked is crack cocaine; due to the efficiency with which cocaine enters the brain, the doses required to be effective are low, while the high obtained is rapid and intense, making it extremely addictive. Cocaine could also be taken intravenously, but this type of intake is not frequent, also because when it is smoked it, nevertheless, reaches high brain concentrations, quite like the intravenous route [7].

Once in the body, cocaine is widely distributed throughout the body and is rapidly metabolized; the main metabolites are benzoylecgonine (BE), ecgonine methyl ester (EME), ecgonine (EC), and norcocaine (NCOC); the latter is highly hepatotoxic and capable of crossing the blood–brain barrier (BBB) [7]. Moreover, in the presence of ethanol, cocaine undergoes a process of transesterification, forming a psychoactive metabolite, cocaethylene, with a biological activity like that of cocaine, which prolongs euphoria and cardiovascular effects; minimal amounts of cocaine are excreted in the parental, non-metabolized, form. The cocaine plasma half-life is around 30 to 90 min, but metabolites are present in the urine for a longer period, from 2 to 5 days.

Cocaine acts as a local anesthetic due to its ability to block neural conduction, but its effects on the central nervous system are due to its ability to inhibit the reuptake of the monoamines norepinephrine (NE), 5-hydroxytriptamine (5-HT), and dopamine (DA) from the synaptic cleft. However, the reinforcing properties of cocaine are mainly due to its effects on DA reuptake in the nucleus accumbens (NaCc) and prefrontal cortex, two important areas of dopaminergic input from the ventral tegmental area (VTA) of the midbrain [6]. The sympathomimetic properties derive instead from the ability of cocaine to block the NE transporter, preventing NE reuptake; the resulting increase in NE concentration leads to increased stimulation of α- and β-adrenergic receptors and sympathetic responses, such as increased pressure and heart rate [7]. Cocaine, however, is also capable of binding to other proteins, such as enzymes, voltage-gated ion channels, plasma proteins, and neurotransmitter receptors, determining clinical effects, mainly on the cardiovascular, respiratory, and renal systems, in addition to those observed on the brain, liver, and muscle [8]. Among its effects, endocrine-disrupting behavior has also been described [9,10], which could pose a serious threat to the development and functioning of the reproductive system in both sexes [11]. Therefore, either because of the increase in cocaine use or the increase in cases of human infertility, in this review, we have analyzed the available literature on the effects of cocaine on animal and human reproduction, highlighting the effects of this drug on the hypothalamic–pituitary–gonadal system, gonads, and reproductive behavior.

## 2. Cocaine and Hypothalamus–Pituitary–Gonadal (HPG) Axis

The hypothalamus–pituitary–gonadal (HPG) axis regulates the activity of gonads, gametogenesis, and reproduction; at the hypothalamic level, the gonadotropin-releasing hormone (GnRH) stimulates gonadotropic cells of the pituitary to produce two gonadotropins, follicle-stimulating hormone (FSH) and luteinizing hormone (LH), which act on the gonads by regulating their activity and inducing the production of gametes and synthesis of sexual hormones, estradiol, progesterone, and testosterone. In addition to exerting their action on different tissues, these hormones help regulate the HPG axis via positive and negative feedback mechanisms. Finally, inhibins, activins, and follistatin help regulate the activity of the HPG axis. Recent studies have shown that this picture is a little more complex because GnRH release is regulated via two hypothalamic neuropeptides with antagonist functions: kisspeptin, located in the preoptic area and in the arcuate nucleus, that stimulates GnRH release; RFamide-related peptide-3 (RFRP-3), the mammalian orthologue of avian gonadotropin-inhibiting hormone (GnIH), located in rodents in the dorsal medial nucleus of the hypothalamus, that inhibits GnRH release, in part, through its action on kisspeptin and/or GnRH neurons [12,13]

The effects of cocaine on the HPG axis have been mainly studied in humans and mammals. Mello et al. (1990), together with other authors in subsequent works [14,15], showed that in female rhesus monkeys during the follicular phase, the acute administration of cocaine rapidly increases the release of the luteinizing hormone (LH), suggesting that this release is mediated via a cocaine-stimulated GnRH release; no effect on FSH release was found. In rhesus monkeys in the luteal phase, a transient increase in circulating LH levels was found, while maximum levels were observed after chronic cocaine administration [16]. Cocaine-stimulated LH release has also been observed in humans of both sexes and in male rhesus monkeys [17,18,19]. Furthermore, an increase in both LH and, to a lesser extent, FSH levels have been found in drug-free human male volunteers following acute cocaine administration [20]. These findings are consistent with those of another study conducted both in vitro and in vivo in female rats [21], which confirmed that cocaine affects LH or FSH release, acting not directly on the pituitary gland, as gonadotropin secretion increases indirectly by increasing hypothalamic GnRH release. In contrast to studies in humans and mammals, chronic exposure to environmental cocaine concentrations decreased the serum levels of FSH and LH in teleost *Anguilla anguilla* [22], probably via the cocaine-stimulated increase in dopamine levels, that in teleost fish inhibits the synthesis and release of gonadotropins [23].

Regarding steroid hormones, increases in plasma estradiol levels were observed in female rhesus monkeys during the follicular but not the luteal phase, whereas no change was observed during both phases in progesterone levels [24]. Conversely, an increase in progesterone levels was found after a single intraperitoneal injection of 15 mg/Kg bw cocaine in rats subjected to sham-adrenalectomy, but not in adrenalectomized rats, evidencing the role of adrenal progesterone in cocaine-induced inhibition of LH release [25]. Many studies reported that cocaine increased plasma testosterone (T) levels only in females but not in male rhesus monkeys [19,26,27], whereas one study performed on humans of both sexes demonstrated lower levels of free T in men who chronically used cocaine and/or opioids, compared with non-users [28]. Berul et al. [29] studied the effects of cocaine on LH and T levels in male rats: a single intraperitoneal injection decreased LH levels over 3 h, while a higher dose caused a significant increase in T serum levels followed by a significant decrease in at least 2 h. In addition, a dose-dependent effect was noted in the chronic administration of cocaine; indeed, when the animals were chronically administered cocaine for 15 days, the low-dose group did not vary significantly from vehicle controls. However, the high-dose group had lower LH and T levels and lower seminal vesicles and epididymis weight; no changes were observed in ventral prostate or testicular weights. All these data taken together suggest that cocaine acts mainly on the hypothalamic–pituitary axis with a possible secondary action at the gonadal level.

## 3. Cocaine and Alterations of Reproductive Behavior

Many studies have examined the relationship between illicit drug use and changes in reproductive behavior, and often the results are contradictory. Psychostimulant drugs such as cocaine have a reputation for increasing sexual interest and sexual activity [30], and speedball (combined use of cocaine and alcohol) has been associated with increased sexual activity [31]; however, no association between cocaine use and sexual desire or sexual activity modifications has also been reported [32,33]. Moreover, as drugs like cocaine have individual effects, often the increase in sexual desire and performance is present in cocaine users that utilize this drug for this specific purpose [34], and, according to Rhodes and Stimson [35], the relationship between drug use and sexual behavior is influenced by a complex interaction between cognition and culture. The use of drugs such as cocaine can lead to a disinhibition of the individual, which, in turn, can interfere with safer sex compliance. Similar conclusions were drawn in a study performed on adult humans of both sexes [36] aimed at identifying a relationship between alcohol consumption, or alcohol and cocaine consumption, and risky sexual behavior, understood as inconsistency in the use of condoms in combination with the presence of multiple partners. The authors concluded that it is too simplistic to establish such an association because sexual human behavior is very complex and influenced by different factors, such as age, work status, history of sexual abuse, and schooling, so it is difficult to prove that drugs are directly responsible for risky sexual behaviors. Similar results were also obtained in female adolescent rats, in which cocaine did not alter sexual motivation or behavior [37]. In contrast, the results of a study [38] performed on healthy cocaine users showed that cocaine-induced dose-dependent increases in sexual desire and decreases in reported condom use likelihood under some conditions, with possible important consequences for the connections between cocaine and sexual risk behavior.

### Cocaine and the Sexual Dysfunction

The results from studies on the effects of cocaine on other aspects of sexual behavior are conflicting; for example, erectile difficulty and/or impotence have been described in association with cocaine use, but also spontaneous erections after intravenous cocaine injections, as well as delayed or premature ejaculation and impaired ejaculatory ability or inhibition of ejaculation [33]. Large amounts of data indicate that dopamine is important for male sexual behavior. Most studies have been performed in mammals, in which dopamine (DA) agonists have been proven to facilitate various aspects of sexual activity in male rats [38], monkeys [39,40], and humans [41]. Cocaine activates dopaminergic mechanisms due to a non-selective block of the reuptake of monoaminergic neurotransmitters [41]. Although cocaine is considered an aphrodisiac, there are no verified experimental data on its effect on sexual behavior in humans or nonhuman primates. The effects of cocaine on sexual behavior were studied in male macaques (*Macaca arctoides*) for 30 min under two different behavioral conditions: low and high baseline sexual activity (partial or complete separation of male and female). In the cocaine-treated group (0.1–1.0 mg/Kg), a suppression of ejaculatory behavior was determined; in fact, a significant reduction in the number of dose-dependent ejaculations was described. This condition was later recovered via the administration of haloperidol (0.003–0.01 mg/Kg), a dopaminergic receptor antagonist. According to the authors, the results obtained suggest that cocaine, at doses tested, suppresses ejaculatory behavior because of dopamine-2 receptor-mediated mechanisms. The cocaine-induced suppression of ejaculatory behavior could be explained by the strong rewarding effect of cocaine alone [42]. However, the dopaminergic function can be increased under experimental conditions. In cocaine-treated rats, sexual activity can be enhanced via sleep deprivation (PSD). These results are likely due to PSD, which causes an increase in DA plasma levels as well as a super sensitivity of dopamine receptors by exacerbating the actions of cocaine on dopaminergic pathways that induce penile erection and ejaculation [43]. The action of cocaine on ejaculation processes has also been studied from the perspective of the receptors used, particularly the gamma-aminobutyric acid (GABA)ergic system. In detail, in a study performed on rats, the ability of GABAergic drugs to be involved in the action of cocaine on the spontaneous genital reflexes (penile-PE erection and ejaculation-EJ) of PSD male rats was investigated. After a 4-day period of PSD, animals were administered GABAergic drugs 1 h before cocaine and placed in observation cages. The administration of GABA-A agonists (muscimol, 2 and 3 mg/Kg sc) reduced the number of animals showing PE, whereas all doses tested of muscimol and bicuculline significantly reduced the frequency of PE. Pre-treatment with the lowest doses of the GABA-B antagonist, phaclofen (1 and 2 mg/Kg sc), also significantly reduced the percentage of rats showing PE; however, after injection of the highest dose, the percentage of animals with PE was very similar to that observed after pretreatment with the vehicle. Both the GABA-B agonist and antagonist significantly reduced the frequency of PE for all doses used compared with the vehicle group. There were no significant differences between control and GABA-A drugs in EJ behavior, while pretreatment with phaclofen 2 mg/Kg increased ejaculatory latency. Considering all these data, the authors hypothesized that GABAergic compounds inhibited PE in PSD male rats and that this inhibition indicated a differential role of GABA receptor subtypes [44]. It is believed that many of the sexual dysfunctions caused by cocaine may be mainly due to hyperprolactinemia; the mechanism responsible for this effect has not yet been fully clarified, although it is believed that it depends on the downregulation of hypothalamic dopaminergic receptors [45]. Finally, decreased sexual satisfaction and enhanced orgasmic ability have been described in females in association with cocaine consumption but also total anorgasmia. Moreover, cocaine appears to amplify atypical sexual practices and seems to affect men and women differently since male cocaine users seem to hold a positive view toward sex, whereas female users express a general dissatisfaction with their sexual situation [33]. Taken together, these results suggested that the major alterations in both male and female sexual behavior induced by cocaine are determined by the interference between this drug and the (GABA)ergic system.

## 4. Cocaine and Changes in Fertility

As evidenced by numerous studies, there is a worrying decline in human fertility to which both genetic and idiopathic factors contribute, as well as problems related to both male and female causes [46]. Experimental evidence shows that drugs can pose a serious risk to fertility. To this end, several observational studies have been conducted, the limit of which, however, is often the difficulty of attributing specific effects to a specific drug due to the heterogeneity of the substances used, variability in dose and frequency of intake, poly abuse frequency, and tendency to engage in risky sexual behavior, which may be a confounding factor. Studies have been performed mainly in humans and mammals and showed that cocaine can affect both male and female fertility.

### 4.1. Males

#### 4.1.1. Effect of Cocaine on Hormones

Large amounts of data accumulated over the past decade have shown that recreational drug use can affect male reproduction inducing both systemic and direct testicular damaging effects. A study conducted in sexually mature male rats showed that cocaine administration not only results in dose-dependent decreases in body weight and locomotor activities but also causes spermatogenesis alteration. The testes are the site of spermatogenesis, an extraordinarily complex process regulated by several factors, both systemic and local, that leads to the differentiation of spermatogonia into spermatozoa. Control factors include the pituitary gonadotropins LH and FSH with an activating role and prolactin (PRL) with an inhibitory function, but also other factors synthesized in the gonad such as the sex hormones testosterone and the major estrogen known as the 17-β estradiol. The levels of these two hormones are strongly correlated since 17-β estradiol is produced via the aromatization of testosterone of the key enzyme P450 aromatase [47,48]. In rats, a daily subcutaneously administration of cocaine hydrochloride (30 or 15 mg/Kg) for a minimum of 72 days induced a decrease in the levels of estrogens (E2), which in males are key hormones in cell differentiation processes, such as spermiohistogenesis [49,50,51,52,53], and at high dose induced an increase in the percentage of spermatozoa having heads separated from the tail [54]. It was assumed that the reduction in E2 levels was due to decreased body weight rather than a direct drug-related effect. However, the decrease in E2 levels justified the significant increase in the T/E2 ratio as well. In contrast, no increases were found in LH and FSH levels after exposure to a 15 mg/Kg dose of cocaine, as well as in the T and PRL levels. Finally, it was shown that cocaine did not affect the weight of the testis or accessory organs, as well as testis histology [54].

#### 4.1.2. Morpho Functional Effects of Cocaine on Germ Cells

Some of the data described in the Section 4.1.1 are in contrast with the results of a more recent work in which a cocaine-induced morpho-functional change in rat testis was demonstrated. Indeed, in the animals exposed to cocaine at different concentrations, DNA isolated from the testis showed a clear dose- and time-dependent fragmentation pattern typical of apoptosis. In detail, the number of apoptotic germ cells increased significantly; in these cells, caspase-3 activity significantly increased in all groups compared with controls (*p* < 0.05). Therefore, the authors hypothesized that exposure to cocaine for 28 days caused significant damage to the male gonad and increased apoptosis in the testes of rats of different ages. Increased caspase-3 activity could be a key pathway related to the early stage of apoptosis as a mechanism of cocaine-induced germ cell loss [55]. In another study conducted by Li et al. [56], a statistically significant increase in germ cell apoptosis was identified as early as 15 days following cocaine injection and persisted up to 90 days, which agreed with the histopathology of cocaine-induced testicular atrophy, suggesting that germ cell death in rat testes after chronic cocaine administration might be, in part, a result of increased apoptosis. Cocaine is rapidly metabolized via two distinct pathways (hydrolytic and oxidative reaction), with less than 5% excreted unchanged in the urine [57]. Apoptosis caused by cocaine exposure can be a result of cocaine itself and/or its metabolites. Furthermore, the cell cycle distribution measured using flow cytometry analysis shows that cocaine exposure can alter cell cycle distribution according to the reduction of S and G2/M phase, and the population of cells in the sub-G1 phase increased as evidence of apoptosis in the three groups after the 28-day cocaine treatment, especially in the 6-week group. Apoptosis, or the disruption of sperm heads, is not the only damage caused by cocaine [58]. Indeed, a study carried out on humans highlighted the influence of cocaine on sperm kinetics. In particular, human sperm samples were treated with 1 to 1000 µM of cocaine hydrochloride for up to 2 h in vitro, and it was shown that after a brief exposure (15 min), the kinematic parameters of sperm movement, straight-line velocity, and linearity decreased in the high concentration groups. However, after longer exposure (2 h) to cocaine, the differences were not significant. In fact, although cocaine binds [59] to sperm membranes with high affinity, the treatment did not alter the intracellular calcium levels of spermatozoa. Moreover, human spermatozoa treated with a high concentration of cocaine were also fully capable of penetrating zone-free hamster oocytes. These results led the authors to hypothesize that cocaine alters sperm motility only at high concentrations following acute exposure [60]. More recently, given the increasingly frequent and chronic use of cocaine, studies on the effects of this drug on spermatogenesis are also shifting to the epigenetic side. Specifically, experiments have been conducted on testes and germ cells of mice, showing the activation of epigenetic marks and mediators following the chronic administration of 10 mg/Kg body weight (bw) of cocaine for 15 days. Chronic cocaine intake in mice altered testicular epigenetic homeostasis by increasing the global levels of methylated cytosine in germ cell and sperm DNA. Cocaine also increased germ cell acetylated histone 3 and 4 and reduced the expression of HDAC1/2 histone deacetylase. Immunolocalization studies showed that HDAC1/2 proteins and acetylated histones 3 and 4 localize to meiotic germ cells. Analysis of mRNA in isolated germ cells shows decreased levels of Hdac1/2/8, Dnmt3b, and Tet1 and increased levels of Dnmt3a upon expression of the Hdac1/2 gene. Taken together, these data led the authors to hypothesize that cocaine intake is associated with testicular toxicity and significant impairments of reproductive functions, expanding the knowledge based on the impact of addictive stimulants on testicular pathophysiology, male fertility, and reproductive health and implying that the disruption of epigenetic homeostasis by cocaine may have potential consequences for future generations [61]. In a more recent study in mice, such epigenetic changes were evidenced directly in sperm. In particular, 272 differentially methylated regions, especially hypomethylated regions upstream of cyclin-dependent kinase inhibitor 1a (Cdkn1a), were identified in the sperm of treated animals, resulting in an influence on the expression of this important gene [62]. In addition, the male offspring of cocaine-experienced mice show a selective increase in Cdkn1a expression in the nucleus compared with female daughters. According to the authors, the intergenerational effects of paternal exposure to cocaine are determined by epigenetic changes in sperm that occur only during spermatogenesis. However, the effects observed in male offspring of cocaine-exposed animals disappeared when mice were subjected to 4 months of drug abstinence before mating [63]. Taken together, these data suggest that cocaine could act on spermatogenesis by bringing about epigenetic changes in the differentiated germ cells without altering the spermatogonia stem cell reserves win [62].

The sperm of cocaine-treated animals, in addition to carrying epigenetic variations, may be a vehicle for cocaine transport. In detail, in a study always performed on mice, sexually mature animals were injected with tritiated cocaine hydrochloride to subsequently localize the radioactivity that was detected in all organs tested, with the highest concentrations per milligram of tissue found in the kidney and sperm within the epididymis. According to the authors, these findings may explain the mechanism underlying male-mediated teratogenesis by hypothesizing that sperm can transport cocaine into the oocyte during fertilization [64].

#### 4.1.3. Effect of Cocaine on Fertilization

The effects of cocaine on fertility and, more directly, on spermatogenesis were investigated in rats. Thirty-day-old male rats of the Sprague Dawley strain were treated with cocaine hydrochloride (15 mg/Kg bw, corresponding to an average single dose for a heavy cocaine user) daily or twice weekly (weekend group, cocaine administered on Saturdays and Sundays) and were mated with female rats with established pregnancies after 100 and 150 days of drug exposure. To evaluate the effects on male gametogenesis, the authors assessed serum levels of testosterone, FSH, and LH in all adult rats, as well as pregnancy rates and litter weights. After 100 days of treatment, rats that received cocaine daily showed a pregnancy rate of only 33% compared with 86% of controls (*p* < 0.05); in rats exposed to cocaine for 150 days, the pregnancy rate was 50% compared with 100% of controls (*p* < 0.05). The birth weight of offspring in the group receiving cocaine every day was 10% lower than in controls (*p* < 0.05). Testis and epididymis weight showed no significant changes compared with controls. However, morphometric analysis showed significant differences between the cocaine-treated rats (both the daily and biweekly cocaine-treated groups) and their respective controls. In particular, the testes showed a statistically significant reduced mean diameter of seminiferous tubule as well as reduced germinal epithelium thickness compared to controls (*p* < 0.05) [65]. In addition, cocaine-exposed animals also showed a higher number of degenerate cells than the controls. Finally, the number of phase VII spermatids was statistically reduced in the two cocaine-treated groups (*p* < 0.05). The data obtained suggested to the authors that chronic administration of cocaine to male prepuberal rats, either continuously or biweekly, has a profound effect on their testicular function, affecting both spermatogenesis and fertility [65]. Therefore, all evidence reported in males suggests that cocaine has detrimental effects on male reproductive processes with both hormonal and tissue effects. Direct alterations to testicular cells have been demonstrated, resulting in increased rates of apoptosis, reduced sperm motility, altered cell cycle, and changes in the DNA methylation profile of gametes.

### 4.2. Females

#### 4.2.1. Effect of Cocaine on Ovary Function

The results of studies performed to understand the relationship between cocaine and female fertility are somewhat contrasting. In a case–control study, based only on self-report, the histories of illicit drug use among infertile women were compared with those of a control group with proven fertility; results showed a possible association of cocaine use with tubal infertility, probably related to pelvic inflammation, widespread among women using cocaine [66]. In contrast, another case–control study showed that cocaine did not affect fertility, but a reduced average time to conception and an increased risk of conceiving were observed in women using cocaine compared to women who had never used it [67]. Studies performed in rats showed that chronic cocaine exposure affected, in a dose-dependent manner, estrous cyclicity that was permanently disrupted at the highest dose (20 mg/Kg bw); also, ovulation rate was reduced together with LH serum levels [15]. Using rhesus monkey females, it has been demonstrated that chronic cocaine intake affected menstrual cyclicity and increased anovulation rate, independent of weight loss, caloric intake, and basal gonadotropin levels [68,69]. In adult female rhesus monkeys, a direct effect of cocaine on ovarian function, probably via the disruption of neural ovarian regulation, was suggested by Thyer et al. [70], who found impaired ovarian responsiveness to exogenous gonadotropins and decreased ovulatory stigma. The altered ovarian function has also been found in rabbits [71] following intravenous cocaine administration (4 mg/Kg bw). In fact, single daily injections during gestation have been shown to induce increased ovulation, probably due to effects on the HPG axis, but did not affect either fetal or placental weight. The authors conclude that probably the perinatal problems associated with human cocaine use are due not only to cocaine use but mainly to a combination of drug use and other risk factors, such as poor nutrition and a lack of prenatal care.

Chen et al. [72] found delayed puberty and slowed body growth and development in juvenile female mice chronically exposed to cocaine. The results obtained from injecting cocaine in preadolescent female Long-Evans hooded rats were quite different [73], as no effects were found on the onset of puberty or the age of the first ovulation, nor on the interval between the first ovulation and the beginning of puberty. However, alterations in estrous cyclicity, a decrease in proestrus transitions, and an increase in the number of cycles lacking clear proestrus transitions were observed, indicative of changes in ovulatory control. Other studies performed in rabbits [74] found that cocaine affects the activity of follicular cells by delaying the luteinization of granulosa cells. Indeed, chronic cocaine decreased the serum and follicular levels of progesterone while increasing the levels of follicular estradiol but did not influence the folliculogenesis stimulated by human chorionic gonadotropin. Overall, the data collected in Section 4.2.1 suggest that cocaine has adverse effects on female gametogenesis, showing the main effects by altering the mechanisms involved in the ovulation process.

#### 4.2.2. Effect of Cocaine on Oocytes Function

In vitro studies have shown that cocaine can disrupt chromosome segregation in mammalian oocytes during meiosis. In particular, mouse oocytes surrounded by the cumulus oophorous were matured in vitro in the continuous presence of cocaine and evaluated for meiotic cell cycle progression and centrosome-microtubule organization using a combination of cytogenetic and fluorescence microscopy techniques. Both approaches showed that cocaine has minimal effects on meiotic cell cycle progression to metaphase II, except at the highest dose tested (1000 µg/mL), where progression from metaphase I to metaphase II was inhibited. Cytogenetic analyses also showed that cocaine treatment impaired the segregation of homologous chromosomes, as well as reduced premature separation of centromeres. Cocaine also caused striking changes in the meiotic spindle structure and cytoplasmic organization of centrosomes. A 36% reduction in spindle length was associated with the loss of nonacetylated microtubules and the fragmentation of spindle pole centrosomes [75]. Alterations in the mitotic spindle structure in oocytes of mice exposed to cocaine have also been shown [76]. To verify the effects of cocaine, the oocytes were exposed in culture to 10, 100, and 1000 µg cocaine/mL for 20 h to the stage equivalent to metaphase II. The spindles were visualized via indirect immunofluorescence staining for tubulin. Concentrations of 10 and 100 mg cocaine/mL had no significant effects on oocyte maturation. However, at a concentration of 10 µg/mL, it had no discernible effect on spindle morphology, while the exposure to 100 µg/mL resulted in a significant increase in the frequency of defective spindles (44 ± 7% compared to 12 ± 2% in controls). The exposure to 1000 µg cocaine/mL also blocked development at the metaphase I stage. The authors highlighted abnormal spindles characterized by barrel shapes and asymmetrical spindle halves containing wavy or branched fibers, as well as chromosomal misalignment was also observed in some cases [76]. Overall, these data showed that cocaine can impair the structure of the meiotic spindle, leading to the incorrect segregation of chromosomes, suggesting that this drug can potentially cause aneuploidies.

### 4.3. No Mammalian Species

#### Effect of Cocaine on Ovary Function

Compared to mammals, there are fewer studies concerning the effects of cocaine on the fertility of no mammalian species. Studies performed in European eels [22] showed that chronic exposure to an environmental cocaine concentration had a profound influence on the ovary, in which a smaller follicular area, a higher amount of connective tissue, and a greater number of previtellogenic oocytes were observed. Also, the presence and the localization of some key enzymes involved in eel oogenesis, 3β-hydroxysteroid dehydrogenase, 17 β-hydroxysteroid dehydrogenase, and P450 aromatase, were affected by cocaine, suggesting a lower maturation rate in ovarian follicles exposed to cocaine, and a potential risk to reproductive health of this species. Studies performed on brown mussels (*Perna perna*) [77] reported an effect of crack cocaine on gametes and embryo-larval development, at high concentrations, in the order of mg/L, not yet reported in aquatic environments. Another study [78] examined the effects of a combination of different doses of crack cocaine in some ocean acidification scenarios on sea urchin (*Echinometra lucunter*) reproduction. The authors found that fertilization rates decreased significantly after short-term exposure to cocaine, in conjunction with a decrease in water pH. Finally, in *Drosophila melanogaster*, chronic cocaine [79] induced defects in the formation and maturation of ovarian follicles and follicle apoptosis and adult lethality, most likely via the variations in levels of serotonin and dopamine.

## 5. Conclusions

**Figure 1:**
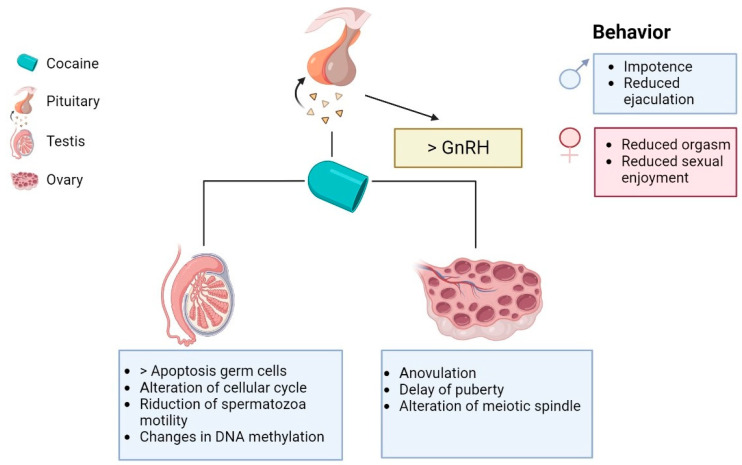
Schematic summary of the potential effects of cocaine on the male and female reproductive systems of mammals.

Reproduction is a strategic moment in an organism’s life, the efficiency of which can be compromised by exposing individuals to exogenous substances, such as drugs. Among the most used drug is cocaine, a plant-derived psychostimulant substance that acts systemically by also affecting reproduction. In the literature, numerous studies conducted mainly in mammals, on both sexes, have shown some effects on both sexual behavior and morpho-functional characteristics of the gonads. Regarding behavioral alterations and changes in the levels of mainly systemic hormones, the available data are rather mixed because the effects depend on several factors, such as the amount of drug administered, the duration of intake, and the route of administration, as well as the fact that chronic cocaine intake may give different effects than sporadic intake in acute form. In addition, the available data are from studies carried out more than 30 years ago, whereby different results obtained could be determined by limitations in the methods used. In contrast, information on the effects on gonadal structure and function is more definite and certain. Indeed, in males, it has been found in model animals that cocaine causes, in addition to an alteration of the normal cell cycle, the apoptosis of germ cells resulting in a reduction in the diameter of seminiferous tubules and a lowering of the seminiferous epithelium (see diagram in Figure 1). An interesting aspect, which at the same time generates concern, is that cocaine affects the DNA methylation of spermatozoa, thus suggesting a possible epigenetic action of this drug, with potential transgenerational effects that need further future investigation. In females, data are more limited and have shown that cocaine causes a delay in puberty when taken at a prepubertal age and causes ovulation failure in sexually mature individuals as well as can alter the mitotic spindle of oocytes (see diagram in Figure 1). In conclusion, although the data available in the literature are not very abundant, in some cases, date back more than 10 years and are somewhat conflicting, all the information gathered in this review points to a number of scientific findings on the potentially detrimental effect of cocaine on testicular and ovarian function, which may support future studies aimed at elucidating the molecular effects of cocaine on reproduction both behaviorally and as a direct effect on the gonads.

## Data Availability

All data analyzed during this study are included in this article.
